# Supplementary material for: Causal Relationship between Aging and Anorexia Nervosa: A White-Matter-Microstructure-Mediated Mendelian Randomization Analysis
Source: Biomedicines. 2024 Aug 16;12(8):1874. doi: 10.3390/biomedicines12081874 (PMC11351342; doi:10.3390/biomedicines12081874)
Supplement: Supplementary file 1 [file biomedicines-12-01874-s001.zip › Supplementary Figure/Figure S4/Singe SNP plot of MR analysis for TL to ubm-b-2120.pdf]

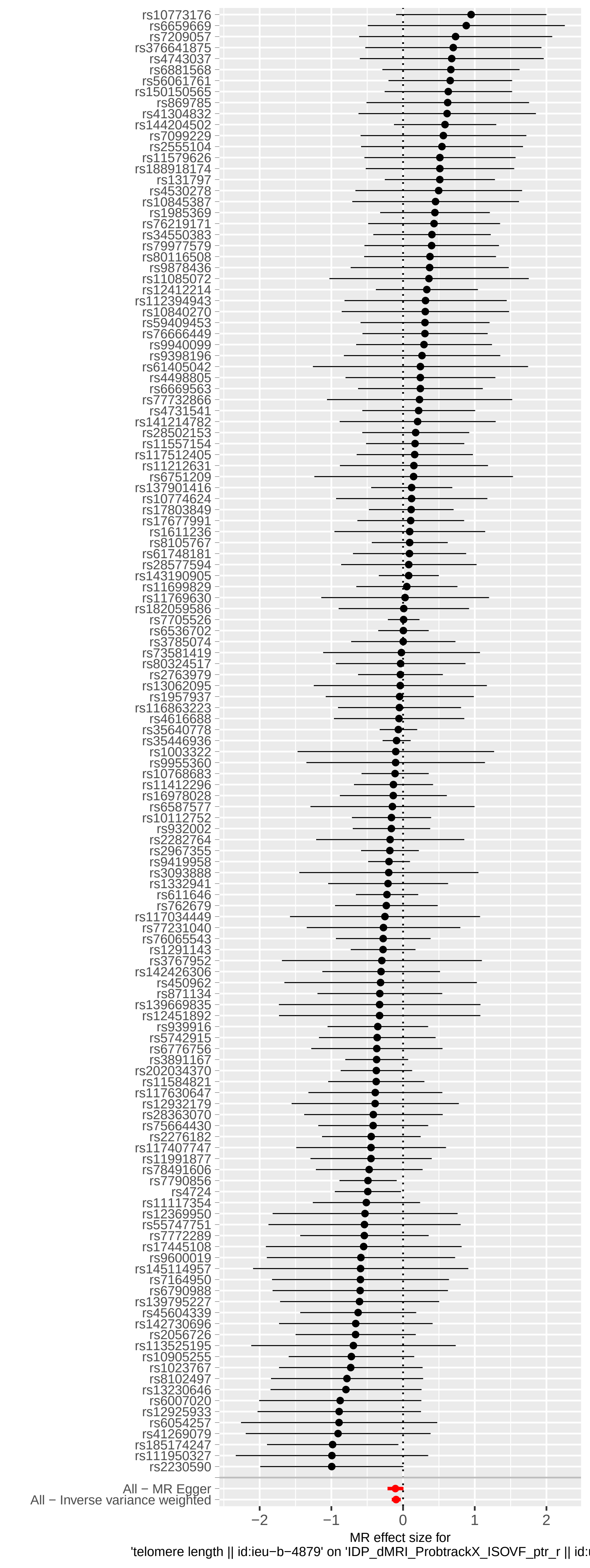
rs10773176
rs6659669
rs7209057
rs376641875
rs4743037
rs6881568
rs56061761
rs150150565
rs869785
rs41304832
rs144204502
rs7099229
rs2555104
rs11579626
rs188918174
rs131797
rs4530278
rs10845387
rs1985369
rs76219171
rs34550383
rs79977579
rs80116508
rs9878436
rs11085072
rs12412214
rs112394943
rs10840270
rs59409453
rs76666449
rs9940099
rs9398196
rs61405042
rs4498805
rs6669563
rs77732866
rs4731541
rs141214782
rs28502153
rs11557154
rs117512405
rs11212631
rs6751209
rs137901416
rs10774624
rs17803849
rs17677991
rs1611236
rs8105767
rs61748181
rs28577594
rs143190905
rs11699829
rs11769630
rs182059586
rs7705526
rs6536702
rs3785074
rs73581419
rs80324517
rs2763979
rs13062095
rs1957937
rs116863223
rs4616688
rs35640778
rs35446936
rs1003322
rs9955360
rs10768683
rs11412296
rs16978028
rs6587577
rs10112752
rs932002
rs2282764
rs2967355
rs9419958
rs3093888
rs1332941
rs611646
rs762679
rs117034449
rs77231040
rs76065543
rs1291143
rs3767952
rs142426306
rs450962
rs871134
rs139669835
rs12451892
rs939916
rs5742915
rs6776756
rs3891167
rs202034370
rs11584821
rs117630647
rs12932179
rs28363070
rs75664430
rs2276182
rs117407747
rs11991877
rs78491606
rs7790856
rs4724
rs11117354
rs12369950
rs55747751
rs7772289
rs17445108
rs9600019
rs145114957
rs7164950
rs6790988
rs139795227
rs45604339
rs142730696
rs2056726
rs113525195
rs10905255
rs1023767
rs8102497
rs13230646
rs6007020
rs12925933
rs6054257
rs41269079
rs185174247
rs111950327
rs2230590
All − MR Egger
All − Inverse variance weighted
−2
−1
0
1
2
MR effect size for
'telomere length || id:ieu−b−4879' on 'IDP_dMRI_ProbtrackX_ISOVF_ptr_r || id:
